# Rab25 promotes erlotinib resistance by activating the β1 integrin/AKT/β‐catenin pathway in NSCLC

**DOI:** 10.1111/cpr.12592

**Published:** 2019-03-07

**Authors:** Jianmin Wang, Pu Zhou, Xudong Wang, Yongxin Yu, Guangkuo Zhu, Linpeng Zheng, Zihan Xu, Feng Li, Qiai You, Qiao Yang, Wenlei Zhuo, Jianguo Sun, Zhengtang Chen

**Affiliations:** ^1^ Institute of Cancer, Xinqiao Hospital Army Medical University Chongqing China

**Keywords:** EGFR‐TKI resistance, NSCLC, Rab25, β1 integrin, β‐catenin

## Abstract

**Objectives:**

Epidermal growth factor receptor tyrosine kinase inhibitor (EGFR‐TKI) has significant therapeutic efficacy in non‐small‐cell lung cancer (NSCLC) patients. However, acquired resistance is inevitable and limits the long‐term efficacy of EGFR‐TKI. Our study aimed to investigate the role of ras‐associated binding protein 25 (Rab25) in mediating EGFR‐TKI resistance in NSCLC.

**Materials and Methods:**

Rab25 expression in NSCLC patients was measured by immunohistochemical staining. Western blotting was used to analyse the expression of molecules in the Rab25, EGFR and Wnt signalling pathways. Lentiviral vectors were constructed to knock in and knock out Rab25. The biological function of Rab25 was demonstrated by cell‐counting kit‐8 and flow cytometry. The interaction between Rab25 and β1 integrin was confirmed by co‐immunoprecipitation.

**Results:**

Rab25 overexpression induced erlotinib resistance, whereas Rab25 knockdown reversed this refractoriness in vitro and in vivo. Moreover, Rab25 interacts with β1 integrin and promotes its trafficking to the cytoplasmic membrane. The membrane‐β1 integrin induced protein kinase B (AKT) phosphorylation and subsequently activated the Wnt/β‐catenin signalling pathway, promoting cell proliferation. Furthermore, high Rab25 expression was associated with poor response to EGFR‐TKI treatment in NSCLC patients.

**Conclusions:**

Rab25 mediates erlotinib resistance by activating the β1 integrin/AKT/β‐catenin signalling pathway. Rab25 may be a predictive biomarker and has potential therapeutic value in NSCLC patients with acquired resistance to EGFR‐TKI.

## 1 INTRODUCTION

Small molecule inhibitors of receptor tyrosine kinases have produced encouraging therapeutic results against non‐small‐cell lung cancer (NSCLC). Erlotinib is an epidermal growth factor receptor tyrosine kinase inhibitor (EGFR‐TKI) that is a first‐line treatment for advanced NSCLC in patients harbouring activated EGFR mutations (ie, exon 19 deletion or exon 21 L858R mutation). According to a randomized phase 3 trial, the median progression‐free survival (PFS) of advanced NSCLC patients was 11 months in the erlotinib group vs 5.5 months in the chemotherapy (gemcitabine/cisplatin) group.^1^

However, almost all patients develop acquired resistance to erlotinib after 6‐12 months of treatment.^2^, ^3^, ^4^ Although a substantial number acquired resistance mechanisms have been identified, such as the T790M mutation and c‐MET amplification, almost 30% of the acquired resistance cases remain unexplainable.^5^ Therefore, appropriate biomarkers that correlate with EGFR‐TKI acquired resistance are urgently needed for better prognostic and therapeutic strategies.

Ras‐associated binding protein 25 (Rab25) is a member of the Rab small GTPase family that regulates intracellular vesicle trafficking.^6^ Membrane trafficking is involved in many cellular pathways related to carcinogenesis, such as cell proliferation, invasion, metastasis and polarity loss.^7^ Jeong^8^ noted that Rab25 upregulates β1 integrin levels and induces snail and fascin expression, leading to epithelial‐mesenchymal transition (EMT) in breast and ovarian cancer cells. Furthermore, Rab25 interacted with α5β1 integrin and regulated the trafficking of this molecule, which promoted tumour progression in ovarian cancer cells.^9^ In our previous study, Rab25 was a candidate oncogene in NSCLC, and its driving role in EGFR‐TKI resistance was investigated.

In our laboratory, we have established erlotinib‐resistant cell lines by exposing cells to increasing doses of erlotinib and did not detect the T790M mutation in the clones.^10^ The Rab25 protein levels were increased in the erlotinib‐resistant cell lines. Furthermore, we observed that Rab25 induced β1 integrin trafficking to the cytoplasmic membrane and stabilized β1 integrin protein to activate the Wnt signalling pathway, ultimately leading to cell survival. Therefore, our study elucidated the mechanism by which Rab25 mediates acquired EGFR‐TKI resistance without the T790M mutation in NSCLC.

## 2 MATERIALS AND METHODS

### 2.1 Patients and samples

Lung cancer specimens were obtained from Xinqiao Hospital, and the protocol was approved by the Medical Ethics Committee of Xinqiao Hospital. Between May 2015 and March 2017, 37 lung adenocarcinoma patients who had received EGFR‐TKI treatment (erlotinib or gefitinib) were enrolled in this retrospective study, and tumour samples were collected. In addition, four tumour samples after acquired resistance to EGFR‐TKI were collected. The clinical response was evaluated according to the Response Evaluation Criteria in Solid Tumors (RECIST) 1.1 criteria. Patients who had progressive disease (PD) or stable disease (SD) for 9 months or less were regarded as EGFR‐TKI poor responders (n = 11). Individuals with a status of complete response (CR), partial response (PR) or SD for more than 9 months were classified as EGFR‐TKI good responders (n = 26). Rab25 protein expression was detected by immunochemistry. In addition, a survival analysis assessing the period from diagnosis to death or study completion (May 2018) was performed. Details of immunohistochemical staining of tumour samples are provided in supporting information.

### 2.2 Western blot and co‐immunoprecipitation analyses

Cells were washed twice with phosphate‐buffered saline (PBS), lysed in radioimmunoprecipitation assay (RIPA) buffer (Beyotime, Shanghai, China) containing a protease inhibitor cocktail (Roche, Basel, Switzerland) for 30 minute and then centrifuged at 13 000 × *g* for 10 minute. After the supernatants were boiled with loading buffer for 10 minute, the samples were loaded onto sodium dodecyl sulphate polyacrylamide gel electrophoresis (SDS‐PAGE) gels and transferred onto polyvinylidene fluoride (PVDF) membranes (Millipore, New Bedford, MA, USA). After blocking, the membranes were incubated separately with specific primary antibodies overnight at 4°C and then incubated with goat anti‐rabbit or goat anti‐mouse immunoglobulin G (IgG) antibodies (ZSGB‐Bio, Beijing, China) for 1 hour at room temperature. Finally, immunoreactive proteins were visualized using a chemiluminescence detection system (FluorChem HD2, Silicon Valley, CA, USA).

For immunoprecipitation (IP) experiments, cells were washed in cold PBS, lysed in a lysis buffer (Beyotime) containing a protease inhibitor cocktail (Roche) for 30 minute and then centrifuged at 13 000 × *g* for 10 minute. The cell lysates were incubated with 1 μg of antibodies overnight at 4°C and then incubated with 30 μL of protein A/G magnetic beads (Bio‐Rad, Hercules, CA, USA) for 2 hour at 4°C. After washing and isolating the beads with a magnet, the beads were boiled for 10 minute in SDS protein loading buffer to elute the bound protein. Subsequently, the IP lysates were assessed by Western blot analysis with the indicated antibodies.

### 2.3 Animal experiments

All animal experiments were approved by the Institutional Animal Care and Use Committee of Xinqiao Hospital. A total of 80 male nude mice (4‐6 weeks old) were obtained from the SPF Laboratory Animal Center of Xinqiao Hospital. A total of 5 × 10^6^ cells were injected into the left armpit of each mouse to establish a xenograft tumour. When tumour volumes reached 100 mm^3^, the mice were randomly allocated into groups of five mice to receive erlotinib by oral gavage at 60 mg/kg for 14 sequential days, with the tumour volumes measured every 3 days. The tumour volume was calculated according to the following equation: *V* = (length × width^2^)/2. After 4 weeks, the mice were sacrificed, and the tumour tissues were fixed and analysed by immunohistochemical staining.

### 2.4 Statistical analysis

All quantitative data are presented as the means ± SEM, and all data from in vitro experiments were obtained from three independent experiments. The data were analysed by one‐way ANOVA or Student's *t* test, and statistical analyses were performed using SPSS21.0 (SPSS, Chicago, IL, USA) and GraphPad Prism 5.0 (GraphPad software, La Jolla, CA, USA). Pearson's chi‐square test was used to compare the relationship between Rab25 expression and the therapy response to EGFR‐TKI. The results were considered to be significant at *P < *0.05. The differences in survival based on the Rab25 expression level were analysed by Kaplan‐Meier (K‐M) survival analysis, and the log‐rank test was used to evaluate the significance of the difference.

## 3 RESULTS

### 3.1 Rab25 is involved in erlotinib resistance

**Figure 1:**
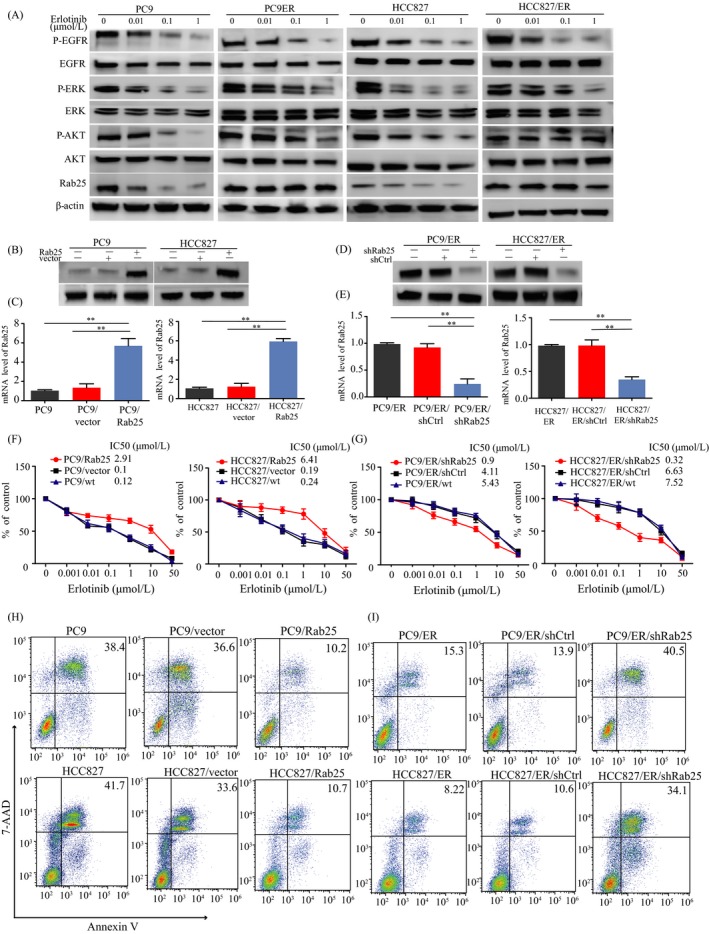
Rab25 mediates erlotinib resistance in lung cancer cells. (A) Western blotting showing the effect of erlotinib on EGFR signalling pathway molecules in the parental and erlotinib‐resistant lung cancer cell lines. Rab25 protein (B) and mRNA (C) expression were analysed by real‐time PCR and Western blotting, respectively, in the PC9 and HCC827 cells transfected with the Rab25‐overexpressing vector and control vector. Western blotting and real‐time PCR analyses of Rab25 protein (D) and mRNA (E) levels after Rab25 knockdown in the PC9/ER and HCC827/ER cells. **P < 0.01, mean ± SEM, Student's *t* test. The effect of Rab25 overexpression in the PC9 and HCC827 cells (F) and the effect of Rab25 knockdown in PC9/ER and HCC827/ER cells (G) on the sensitivity of the cells to erlotinib were measured using a CCK‐8 assay. (H, I) Erlotinib‐mediated cell apoptosis in the indicated cells was detected by flow cytometry

We previously observed that Rab25 expression was higher in NSCLC tissue than in adjacent normal tissue, and high Rab25 expression was significantly correlated to worse prognosis.^11^ To further explore the role of Rab25 in EGFR‐TKI resistance, we examined the expression of Rab25 and EGFR signalling in the EGFR‐TKI‐sensitive PC9 and HCC827 cells and the resistant PC9/ER and HCC827/ER subclones. The phosphorylation of EGFR and ERK (p‐EGFR and p‐ERK) was inhibited by erlotinib in a dose‐dependent manner in the four cell lines (Figure 1A). However, the p‐AKT and Rab25 levels were only decreased in the sensitive cells, as p‐AKT and Rab25 expression could be detected in the presence of high concentrations of erlotinib in the resistant cells (Figure 1A). To further elucidate whether Rab25 affects cell sensitivity to erlotinib, we generated PC9/Rab25 and HCC827/Rab25 cells by transfecting the parental cell lines with Rab25 cDNA (Figure 1B,C), and the PC9/Rab25 cells showed an obvious resistance to erlotinib (Figure 1F). Consistent with the variation in sensitivity, the PC9/Rab25 and HCC827/Rab25 cells exhibited decreased apoptosis in response to erlotinib intervention compared with the corresponding parental cells (Figure 1H). Moreover, Rab25‐knockdown PC9/ER and HCC827/ER cells (Figure 1D,E) exhibited increased sensitivity to erlotinib (Figure 1G) and greater numbers of apoptotic cells (Figure 1I).

### 3.2 Wnt signalling is correlated with Rab25‐mediated erlotinib resistance

**Figure 2:**
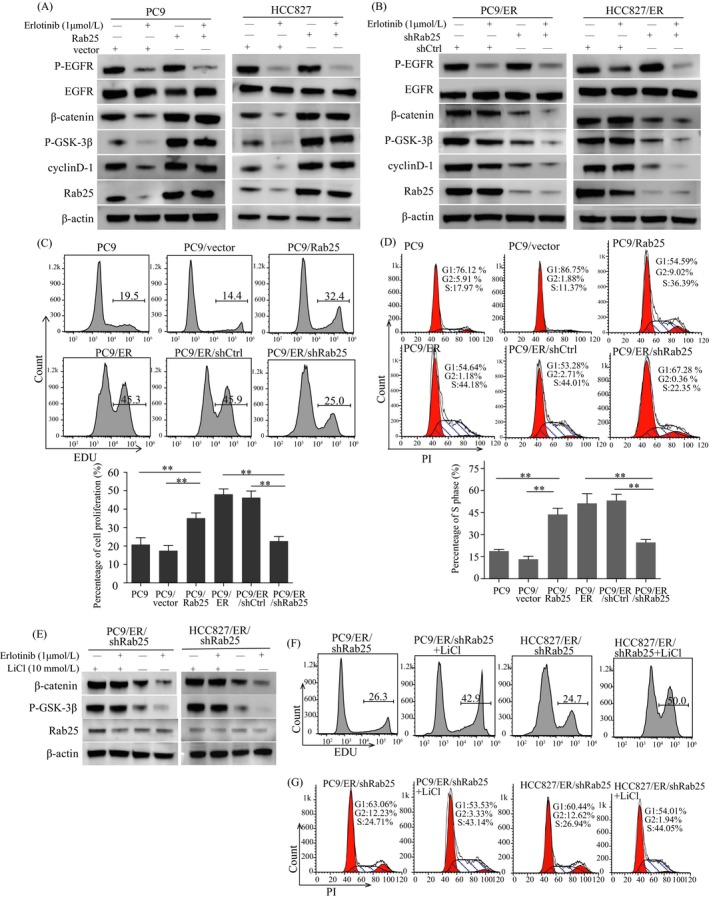
The Wnt signalling pathway is involved in Rab25‐mediated erlotinib resistance. (A, B) Western blotting showing key molecules of the Wnt signalling pathway in the indicated cells. (C) Cell proliferation in the indicated cells was measured by an EdU assay. (D) Propidium iodide staining detected cell cycling in the indicated cells, and ModFit was used to analyse the S phase ratio of the cells. **P < 0.01, mean ± SEM, ANOVA. After treatment with LiCl for 24 h, the P‐GSK‐3β and β‐catenin levels were measured in the PC9/ER/shRab25 and HCC827/ER/shRab25 cells by Western blotting (E), and cell proliferation (F) and cell cycling (G) were detected in these cells.

In our previous study, the Wnt signalling pathway was observed to be closely associated with lung adenocarcinoma (data not shown). Rab25 has been reported to activate the Wnt pathway and promote cell proliferation.^12^, ^13^ GSK‐3β, a crucial molecule in the Wnt pathway, can be phosphorylated by AKT.^14^ Interestingly, erlotinib was observed to inhibit p‐EGFR and p‐ERK expression but not p‐AKT or Rab25 expression in the PC9/ER and HCC827/ER cells (Figure 1A). Therefore, we speculated that Rab25 was associated with the observed AKT activation and the stimulation of the Wnt pathway. We observed that Rab25 overexpression induced the expression of crucial molecules in the Wnt pathway (p‐GSK‐3β and β‐catenin) and facilitated erlotinib resistance in the PC9 and HCC827 cells (Figure 2A). In addition, Rab25 knockdown led to the inhibition of these proteins and erlotinib re‐sensitization in the PC9/ER and HCC827/ER cells (Figure 2B). The activation of the Wnt pathway resulted in cell cycle transitions from G1 to S phase, promoting cell proliferation. As a result, Rab25 overexpression increased the percentage of cells in S phase and that of EdU‐positive cells (Figure 2C,D, and Figure S1A,B). Rab25 knockdown had the opposite effect. Moreover, LiCl, a Wnt signalling agonist, abolished the Wnt signalling inhibition induced by Rab25 knockdown in the PC9/ER and HCC827/ER cells (Figure 2E) and promoted cell proliferation and G1‐S phase progression (Figure 2F,G). These results suggest that Rab25 activated the Wnt signalling pathway to induce erlotinib resistance.

### 3.3 β1 integrin‐mediated activation of the Wnt signalling pathway

**Figure 3:**
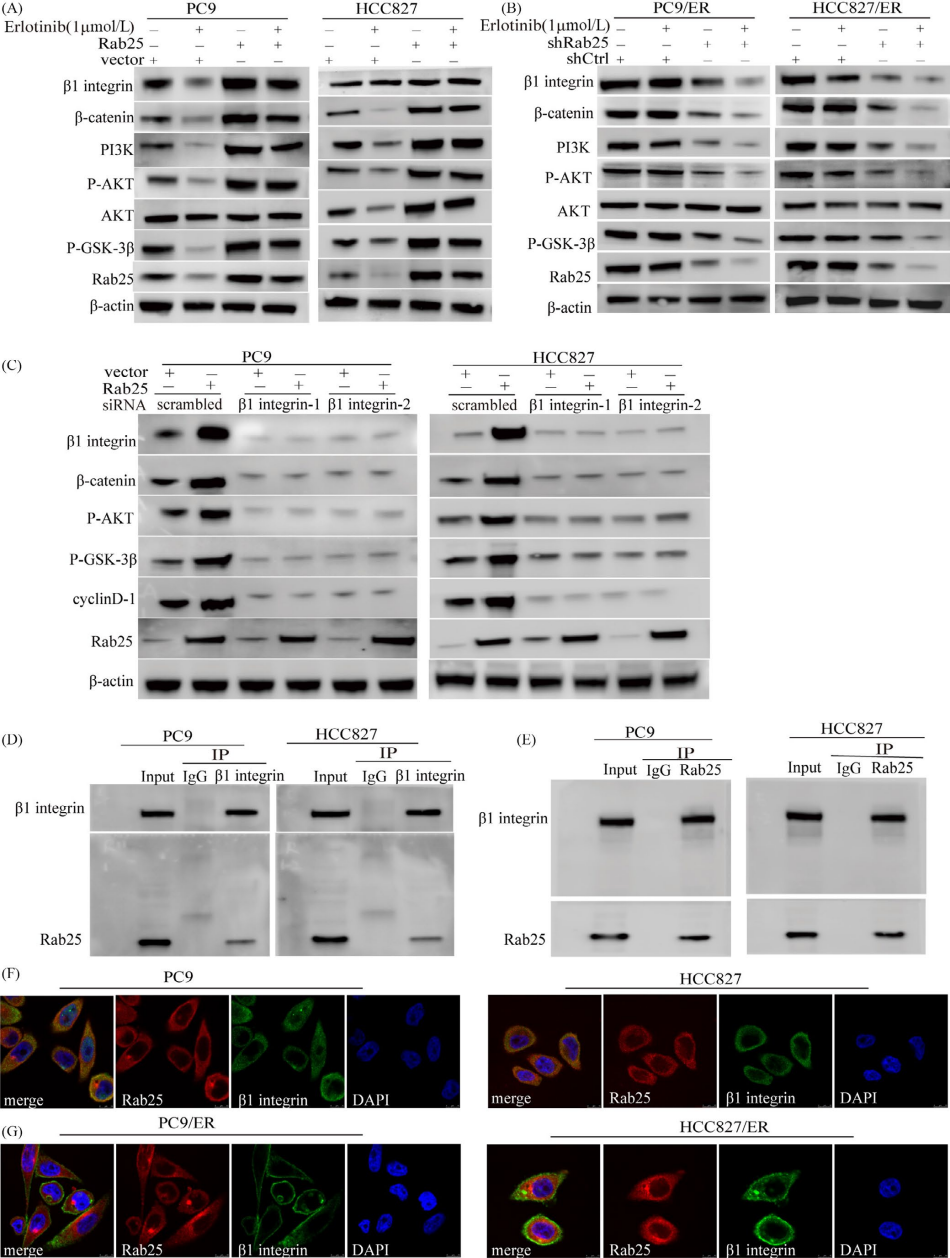
β1 integrin mediates the Rab25‐induced Wnt signalling activation. Western blotting analysis showing the effects of Rab25 overexpression on β1 integrin, p‐GSK‐3β, β‐catenin, PI3K and p‐AKT expression in the PC9 and HCC827 cells (A), and the effects of Rab25 knockdown on these molecules in the PC9/ER and HCC827/ER cells (B). (C) PC9 and HCC827 cells were transfected with the Rab25 overexpression lentiviral vector and β1 integrin interfering RNA, and then, the p‐GSK‐3β, β‐catenin, cyclinD‐1 and p‐AKT levels were measured by Western blotting. Cell lysates from PC9 and HCC827 cells were subjected to immunoprecipitation with anti‐β1 integrin (D) and anti‐Rab25 (E) antibodies, and then, the immunoprecipitates were assessed using the indicated antibodies. (F) Cytoplasmic Rab25 colocalized β1 integrin in the PC9 and HCC827 cells treated with erlotinib. (G) β1 integrin recycling to the plasma membrane in the PC9/ER and HCC827/ER cells treated with erlotinib. Protein localization was detected by immunofluorescent staining and observed by confocal microscopy. Scale bars, 10 μm

Rab25 was reported to regulate integrin‐recycling vesicle trafficking to the cytoplasmic membrane. As an important member of the integrin family, β1 integrin activates PI3K/AKT and regulatory proteins.^15^ Rab25 overexpression induced β1 integrin and p‐AKT expression in the PC9 and HCC827 cells (Figure 3A). In contrast, Rab25 knockdown led to a decrease in these proteins, and the effects were enhanced by erlotinib treatment in the PC9/ER and HCC827/ER cells (Figure 3B). In addition, silencing β1 integrin expression reduced Rab25‐mediated p‐AKT, p‐GSK‐3β and β‐catenin expression, suggesting that β1 integrin is important for Rab25‐induced Wnt signalling activation (Figure 3C). As shown in Figure 3D,E, the results of a co‐IP assay confirmed the direct interaction between Rab25 and β1 integrin. Upon examination by confocal microscopy, we observed that Rab25 colocalized with β1 integrin in the cytoplasm of the PC9 and HCC827 parental cells in the presence of erlotinib (Figure 3F). However, the increased recycling of β1 integrin from the cytosol to the cell surface was observed in the PC9/ER and HCC827/ER cells (Figure 3G). In combination, these results suggest that the Rab25‐mediated regulation of β1 integrin recycling was responsible for activating the Wnt pathway and inducing erlotinib resistance.

### 3.4 Rab25 induces erlotinib resistance in vivo

**Figure 4:**
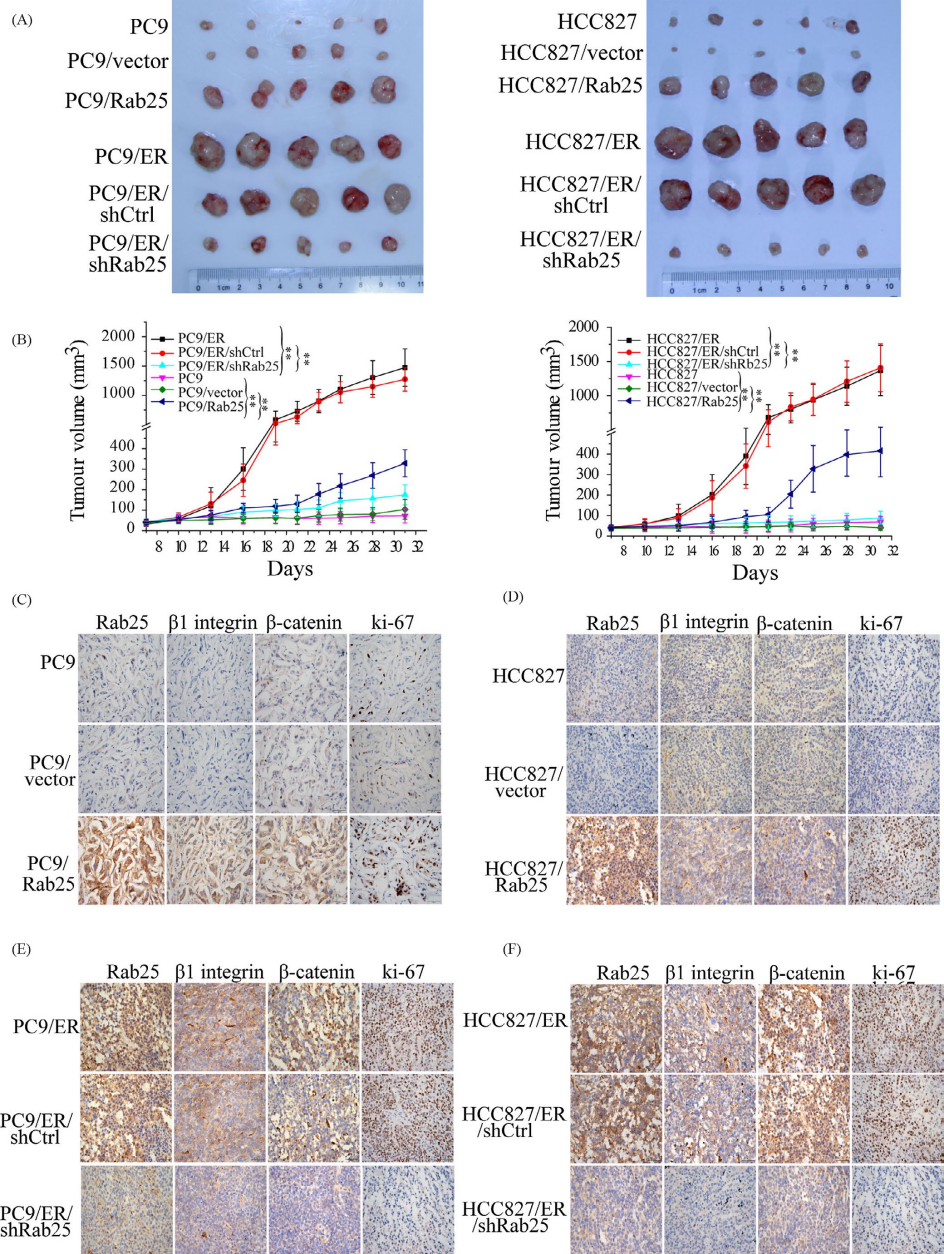
Rab25 mediates erlotinib resistance in lung cancer in vivo. (A) Mice were implanted subcutaneously with the indicated cell lines (5 × 10^6^), and tumours were grown to ~100 mm^3^ and then treated with erlotinib for 14 d. Subsequently, the mice were euthanized, and the tumours were dissected. (B) Tumour growth curve representing the mean ± SEM of the tumour volumes of five mice in the indicated xenograft groups. **P < 0.01, one‐way ANOVA. (C‐F) Immunohistochemical staining assay to confirm the expression of Rab25, β1 integrin and β‐catenin in the indicated group of tumour samples. Tumour cell proliferation was measured by Ki‐67 staining. Scale bars, 50 μm

To further assess the ability of Rab25 to affect the erlotinib response in vivo, stable Rab25‐overexpressing cells (PC9/Rab25 and HCC827/Rab25) were subcutaneously injected into nude mice followed by erlotinib treatment. Compared to the tumours in the control group, those in the PC9/Rab25 and HCC827/Rab25 groups exhibited significantly larger tumour volumes after erlotinib treatment for 14 days (Figure 4A,B). Moreover, mice injected with stable Rab25‐depleted cells (PC9/ER/shRab25 and HCC827/ER/shRab25) showed smaller tumour volumes than did the PC9/ER/shctrl‐ and HCC827/ER/shctrl‐injected mice (Figure 4A,B). Furthermore, the immunohistochemical analysis results showed higher Rab25, β1 integrin, β‐catenin and ki‐67 expression in tumour tissues from the PC9/Rab25 and HCC827/Rab25 groups than those observed in the PC9/vector and HCC827/vector groups (Figure 4C,D). Consistent with the tumour volume data, the tumour tissue samples from the PC9/ER/shRab25 and HCC827/ER/shRab25 groups expressed lower Rab25, β1 integrin, β‐catenin and ki‐67 protein levels than those from the PC9/ER/shctrl and HCC827/ER/shctrl groups (Figure 4E,F). These results demonstrate that Rab25/β1 integrin/β‐catenin signalling is crucial for erlotinib resistance in vitro and in vivo.

### 3.5 Elevated Rab25 expression is associated with EGFR‐TKI resistance

**Figure 5:**
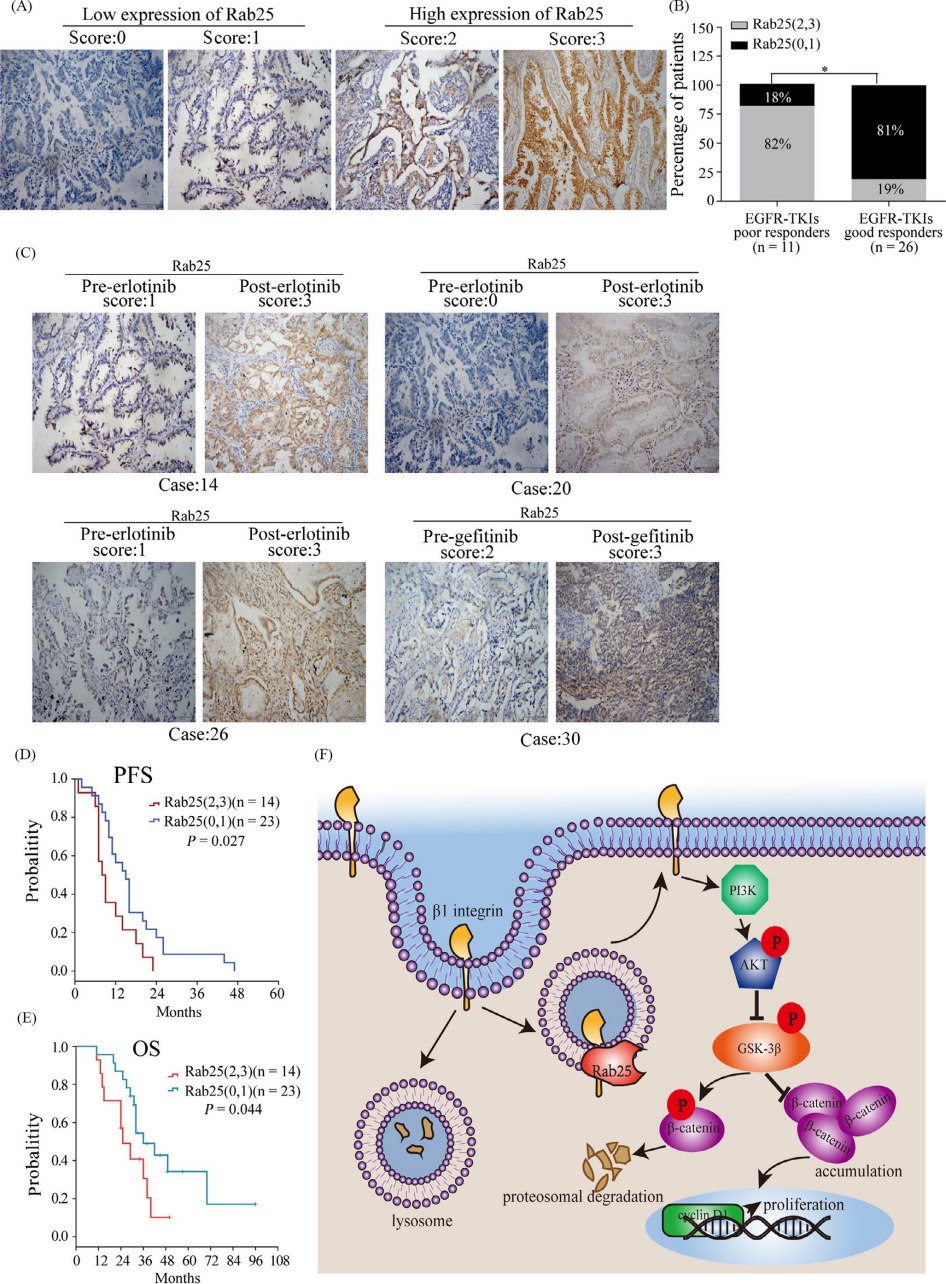
Rab25 expression is associated with the therapeutic response to EGFR‐TKI treatment in NSCLC. (A) IHC staining analysis of Rab25 expression in NSCLC patient tissue samples. A score of 0‐1 indicates low expression of Rab25, and a score of 2‐3 indicates high expression. (B) Patients with high expression (grey bar) and low expression (black bar) were assigned based on their therapeutic responses to EGFR‐TKI. **P < 0.01, Pearson's chi‐square test. (C) IHC staining analysis of Rab25 expression in tissue samples from patients who had acquired resistance to erlotinib or gefitinib. Scale bars, 100 μm. (D‐E) Kaplan‐Meier analysis of progression‐free survival and overall survival in NSCLC patients. (F) Graphical model for the mechanism by which Rab25 regulates β1 integrin/AKT/β‐catenin signalling pathway

Finally, we examined whether Rab25 expression may be correlated with EGFR‐TKI resistance in NSCLC. We retrospectively collected specimens from 37 NSCLC patients who had received EGFR‐TKI therapy and detected Rab25 expression by IHC staining. The clinicopathologic features of these patients are summarized in Table 1. Most patients who had a poor response to EGFR‐TKI treatment exhibited high Rab25 protein levels (Figure 5A,B), and this group of patients had shorter PFS and OS than their counterparts with low levels of Rab25 (PFS of 10 months vs 16 months, OS of 28 months vs 49 months, respectively) (Figure 5D‐F). In addition, Rab25 expression was increased in the acquired resistance to erlotinib or gefitinib tumour samples compared to the initial specimens (Figure 5C). These findings indicate that Rab25 may be involved in EGFR‐TKI in NSCLC.

**Table 1:** Correlation of Rab25 expression and clinicopathological factors of lung adenocarcinoma patients

Clinicopathological features	Rab25 expression		*P*‐value
	Rab25(0,1)	Rab25(2,3)	
Age(median age = 58 y)			
≥58	13	8	0.956
<58	10	6	
Gender			
Female	14	6	0.286
Male	9	8	
Smoking			
Yes	8	2	0.327
No	15	12	
TNM stages			
Ⅲstage	7	5	0.45
Ⅳstage	16	9	
EGFR‐TKIs			
Erlotinib	10	6	0.995
Gefitinib	8	5	
Icotinib	3	2	
EGFR mutation sites			
19del	16	9	1.0
21L858R	7	5	

## 4 DISCUSSION

In the last decade, EGFR‐TKI has been a milestone therapy in NSCLC patients harbouring EGFR‐sensitive mutations. However, acquired resistance over a short or long period of time is inevitable. Although the T790M mutation is the most common cause of resistance (40%‐55%),^16^ the remaining large proportion of relapsed patients exhibit a variety of other triggers, that is, mutations in MET, KRAS and PIK3CA.^17^ Moreover, some unelucidated mechanisms of EGFR‐TKI resistance remain unknown.

Rab25, together with Rab11a and Rab11b, belongs to the Rab11 family. Rab25 participates in apical recycling and transcytosis to sustain polarity in epithelial cells.^6^ Since the loss of cell polarity is an essential characteristic of cancer, Rab25 has been well studied in cancers. As an oncogene or tumour suppressor, the reported roles of Rab25 are diverse in existing publications. In ovarian cancer,^12^ breast cancer^18^ and lung cancer,^19^ Rab25 is reported to enhance tumour progression. However, Rab25 is regarded as a tumour suppressor in colon cancer.^20^ In our previous study, Rab25 protein was high in lung cancer tissue, and it was significantly correlated with clinical progression.^6^ Thus, we considered that Rab25 promotes tumour growth in NSCLC. In this study, we explored the role of Rab25 in EGFR‐TKI resistance, observing that high Rab25 expression is in erlotinib‐resistant cells. Moreover, this resistance is partially reversed by Rab25 silencing, whereas Rab25 overexpression could induce erlotinib resistance in vitro and in vivo. Next, we assessed Rab25 expression in NSCLC patients who received EGFR‐TKI treatment and observed that patients with low Rab25 expression had a higher probability of responding to EGFR‐TKI therapy and better PFS and OS outcomes. These results suggest that Rab25 can negatively regulate the antitumour effect of EGFR‐TKI and is a biomarker for predicting the curative effect of EGFR‐TKI therapy in NSCLC.

Rab GTPases mediate vesicle trafficking between organelles, including the endosomal trafficking and subcellular localization of some integrins.^7^ Integrins participate in signal transduction from the outside to the inside of cells and regulate several biological processes, including cell adhesion, cell migration and tumour progression. Furthermore, integrins regulate several intracellular signalling pathways by recruiting and activating kinases and signalling adaptors.^21^ β1 integrin is the most common component of the integrin family and is overexpressed in numerous cancers. β1 integrin is composed of extracellular, transmembrane and cytoplasmic domains. The cytoplasmic domain is coupled to the cytoskeleton and some downstream signalling molecules, such as tyrosine kinase and serine‐threonine kinase, which can promote tumour attachment, proliferation, invasion and migration.^22^, ^23^ B. Tian and T. Shibue reported that β1 integrin activates FAK and PI3K/AKT and promotes cell survival.^15^, ^24^ In our erlotinib‐resistant cell lines, AKT phosphorylation was also observed with continuous erlotinib treatment. However, β1 integrin silencing reduced the AKT phosphorylation. Even so, the mechanism of β1 integrin‐mediated phosphorylation of some kinases is still not fully understood. The results of a previous study revealed that Rab25 recycles β1 integrin from lysosomal degradation to the plasma membrane, extending the biological role of the recycled protein.^25^ BY Jeong reported that Rab25 induces β1 integrin expression, which activates the EGFR/VEGFA/VEGFR1/snail signalling axis, leading to EMT in ovarian cancer cells.^8^ Consistent with previous data, we observed that Rab25 associated with β1 integrin and mediated β1 integrin recycling to the plasma membrane to participate in biological activities. As a result, it is highly probable that Rab25 mediates erlotinib resistance by mediating the recycling of β1 integrin to the cytoplasmic membrane, which leads to AKT phosphorylation and subsequent downstream signalling to promote tumour cell proliferation.

Aberrant Wnt pathway signalling and active Wnt‐targeted genes are observed in many cancers.^26^ β‐catenin is a key component of the Wnt signalling cascade and is involved in cell proliferation and tumour progression by causing the transcription of several genes, that is, cyclin‐D1 and c‐myc.^27^ The results of previous studies have revealed that the level of β‐catenin is increased in NSCLC cell lines with EGFR‐TKI resistance and that inhibiting β‐catenin signalling enhances sensitivity to EGFR‐TKI treatment.^28^ These results are consistent with our observations, which showed that β‐catenin levels increased in erlotinib‐resistant cell lines and that the change resulted in cell proliferation and resistance maintenance. Crosstalk between integrins and the Wnt/β‐catenin signalling pathway was noted in a number of studies. Lin^29^ reported that β5 integrin interacts with β‐catenin, enhancing the stability of β‐catenin and activating the Wnt pathway. In addition, α6A integrin regulated the Wnt pathway through phosphorylation of β‐catenin.^30^ Consistent with these results, we observed that the β1 integrin‐driven phosphorylation of AKT caused the inactivation of phosphorylated GSK‐3β, resulting in β‐catenin accumulation and leading to cell proliferation in the erlotinib‐resistant cell lines. Thus, the results of the present study suggest that Rab25 induced erlotinib resistance via the β1 integrin/AKT/β‐catenin pathway.

In summary, the results of our study indicate that Rab25 facilitates erlotinib resistance by mediating β1 integrin trafficking to the cytoplasmic membrane, inducing AKT phosphorylation and activating the Wnt/β‐catenin signalling pathway, resulting in cell proliferation. Thus, Rab25 may be an independent predictive biomarker for EGFR‐TKI treatment, and the combination of Rab25 and EGFR inhibitors may be a potential therapeutic mode to reverse resistance to EGFR‐TKI therapy.

## CONFLICT OF INTEREST

All authors declared no conflicts of interest.

## AUTHORS’ CONTRIBUTIONS

JW, PZ, GZ, LZ, ZX and XW carried out the experiments. PZ, YY and QY enrolled patients and collected the patients’ tumour samples. ZX, QY, FL and XW analysed the data. WZ, JS and ZC conceived and designed the experiments. JW and GZ wrote the manuscript. All authors read and approved the final manuscript.
