# Supplementary material for: Rab25 promotes erlotinib resistance by activating the β1 integrin/AKT/β‐catenin pathway in NSCLC
Source: Cell Prolif. 2019 Mar 7;52(3):e12592. doi: 10.1111/cpr.12592 (PMC6536583; doi:10.1111/cpr.12592)
Supplement: Supplementary file 2 [file CPR-52-e12592-s002.docx]

**Supporting Information**

**Materials and Methods**

**Cell culture and reagents**

The human lung adenocarcinoma cell line PC9 was purchased from the Shanghai Institute of Biochemistry and Cell Biology (Shanghai, China), and HCC827 cells were obtained from the American Type Culture Collection. Both cell lines were cultured in RPMI 1640 medium supplemented with 10% foetal bovine serum and 1% penicillin/streptomycin at 37 °C in a humidified 5% CO_2_ atmosphere. To generate erlotinib-resistant cell lines, the PC9 and HCC827 cells were exposed to high-doses (1-10 μM) of erlotinib for a short time, after which the surviving cells were continuously exposed to low doses of erlotinib (0.1 μM). This alternative erlotinib administration approach was performed for 8 months to establish resistant cell lines, PC9/ER and HCC827/ER. Additionally, the two resistant cell lines do not have a secondary T790M mutation.

Erlotinib was obtained from SelleckChem (USA), and LiCl was purchased from Sigma-Aldrich (USA). Rabbit antibodies against EGFR, p-EGFR, protein kinase B (AKT), p-AKT, phosphoinositide 3-kinase (PI3K), extracellular signal-regulated kinase (ERK), p-ERK, Rab25, glycogen synthase kinase (GSK), and β-catenin, and cyclin D1 and a mouse antibody against Ki-67 were purchased from Cell Signaling Technology (USA). An anti-mouse β1 integrin antibody was purchased from R&D Systems (USA), and an anti-rabbit β-actin antibody was obtained from ZSGB-Bio (China).

**Immunohistochemistry**

Formalin-fixed, paraffin-embedded tissue sections were dewaxed in xylene and hydrated in a series of graded concentrations of ethanol, after which the sections were subjected to antigen retrieval by heating at 95 °C in citrate or Tris-EDTA buffer. The sections were incubated with primary antibodies at 4 °C overnight followed by biotinylated secondary antibodies at room temperature for 1 h and a 30 min incubation with horseradish peroxidase-conjugated streptavidin. Immunostaining was performed with 3,3’-diaminobenzidine (DAB) for 3 min, after which the cells were counterstained with haematoxylin. The immunohistochemistry (IHC) staining analysis was performed by two independent pathologists. The immunostaining intensity was scored according to the percentage of positive cells with the following criteria: score 0, no or weak staining in < 5% of cells; score 1, weak staining in 5-25% of cells; score 2, moderate staining in 26-50% of cells; and score 3, strong staining in > 50% of cells. The expression levels classified the samples into two groups according to the IHC scoring: the low-expression group (score 0 and 1) and the high-expression group (score 2 and 3). The IHC analysis was performed using the primary antibodies anti-rabbit Rab25 (1:50), anti-mouse β1 integrin (1:100), anti-β catenin (1:200), anti-cyclin D1 (1:200), and anti-Ki-67 (1:400).

**Immunofluorescence**

After cell fixation with 4% formaldehyde, permeabilization with 0.5% Triton and blocking with 5% bovine serum albumin (BSA), immunofluorescent staining was performed with anti-Rab25 (1:50) and anti-β1 integrin (1:100) antibodies at 4 °C overnight. Next, the cells were incubated with Alexa Fluor 488-conjugated goat anti-mouse IgG (1:200) and Alexa Fluor 647-conjugated goat anti-rabbit IgG (1:200) antibodies for 1 h in the dark. The cell nuclei were counterstained with DAPI, and the cells were imaged with a laser scanning confocal fluorescence microscope.

**Lentiviral vector and siRNA transfection**

The lentiviral vector for RNA interference of Rab25 was purchased from GeneChem (China), and the Rab25 overexpression lentiviral vector was purchased from HanBio (China). The RNAi sequence was GGAAGACCAATCTACTCTC, and the oligonucleotides were ligated with the hU6-MCS-Ubiquitin-EGFP-IRES-Puro vector. Full-length human Rab25 was PCR amplified and cloned into the vector pCDH-CMV-MCS-EF1-GFP-T2A-Puro. Next, the constructed plasmids or the control vector were transfected into HEK-293 cells, and the viruses were subsequently harvested. Cultured cells were infected with 5 µg/ml Polybrene and lentivirus (MOI = 100) for 24 h, after which the cells were incubated with fresh medium for an additional 48 h to establish stable cell lines.

The siRNA against β1 integrin and the scrambled siRNA were purchased from GeneBio (China), and the short-hairpin RNA sequences were si-β1 integrin -1:GGAACCCUUGCACAAGUGA and si-β1 integrin-2: GGCGUAACAAUAAGUUACGAA. Cultured cells were transiently transfected according to the manufacturer’s instructions (RiboBio, China).

**RNA isolation and quantitative real-time PCR (qPCR)**

Total RNA was extracted using TRIzol Reagent (Invitrogen, USA) following the manufacturer’s instructions. After the concentration and purity of the total RNA was determined, reverse transcription was performed using a PrimeScript RT reagent kit (TaKaRa, Dalian, China). For qPCR analysis, the cDNA was amplified with a SYBRPremix Ex Taq (TaKaRa) kit by using an AB 7500 Real-time PCR system. The relative gene expression was calculated using the 2^−ΔΔ^Ct method. The following primers were used: Rab25 (Forward 5’-GTCGTCATGCTCGTGGGTA-3’, Reverse 5’-GACAGTCTCAAAGGCTAGCTCA-3’) and β-actin (Forward 5’-GCGAGCACAGAGCCTCGCCTT-3’, Reverse 5’-CATCATCCATGGTGAGCTGGCGG-3’). The Rab25 mRNA expression levels were normalized to those of β-actin.

**CCK-8 assay for cell viability**

Cells (5×10^3^) were seeded into 96-well plates for 24 h, after which the cells were treated with erlotinib at concentrations ranging from 0.01 to 100 µM for 48 h. Cell counting kit-8(CCK-8) reagent was added to each well, and the cells were incubated for 1 h. Subsequently, the absorbance of the samples was measured at 450 nm using a spectrophotometer (Epoch; BioTek, USA). The percentage of viable cells was reported as the relative absorbance normalized to that of the untreated cells.

**Flow cytometry analysis for cell cycle, apoptosis and proliferation**

For the cell cycle analysis, after being treated with erlotinib, cells were fixed in ice-cold 70% ethanol overnight at 4 °C. Next, the fixed cells were stained with s 50 μg/ml propidium iodide (PI) solution (Beyotime, China) containing 200 mg/ml RNase for 30 min and then were analysed with a flow cytometer (Beckman Coulter, USA).

For the apoptosis analysis, the cells were incubated with erlotinib, stained with anti-annexinV/7-AAD antibodies in binding buffer for 15 min and then analysed with a flow cytometer.

A 5-ethynyl-2'-deoxyuridine (EdU) assay (GeneCopoeia, USA) was performed to evaluate cell proliferation. After erlotinib treatment, cells were incubated with 30 μM EdU, and then, fixation, permeabilization, and staining were performed according to the manufacturer’s instructions. The percentages of proliferating cells were analysed by flow cytometry.
